# Micro-Fibrillated Cellulose in Adhesive Systems for the Production of Wood-Based Panels

**DOI:** 10.3390/molecules25204846

**Published:** 2020-10-21

**Authors:** Emmanouil Karagiannidis, Charles Markessini, Eleftheria Athanassiadou

**Affiliations:** CHIMAR HELLAS S.A.,15 km National Road, Thessaloniki–Polygyros, 57001 Thessaloniki, Greece; CharlesM@ari.gr (C.M.); eathan@ari.gr (E.A.)

**Keywords:** micro-fibrillated cellulose, formaldehyde adhesives, wood-based panels

## Abstract

Micro-Fibrillated Cellulose (MFC) is a new type of bio-based additive, coming from wood cellulose. It can compete and substitute oil derived chemicals in several application fields. In the present work, the use of micro-fibrillated cellulose, in waterborne adhesive systems applied in the manufacture of composite wood-based panels was evaluated. Research was conducted to test the potential of improving the performance of wood-based panel types such as particleboard, waferboard or randomly-oriented strand board and plywood, by the application of MFC and the substitution of conventional and non-renewable chemical compounds. The approaches followed to introduce MFC into the adhesive systems were three, i.e., MFC 2% suspension added during the adhesive resin synthesis, MFC 10% paste admixed with the already prepared adhesive resin and MFC 2% suspension admixed with the already prepared resin. It was found that MFC improves not only the performance of the final wood panel products but also the behaviour of the applied adhesive polymer colloids (e.g., rheology improvement), especially when admixed with the already prepared resins. Moreover, it was proven that when MFC is introduced into the adhesive resin system, there is a possibility of decreasing the resin consumption, by maintaining the board performance. MFC’s robustness to pH, shear and temperature makes it a highly interesting new additive for adhesive producers. In addition, its natural origin can give adhesive producers the opportunity to move over to more environmentally friendly product solutions.

## 1. Introduction

Cellulose is the most abundant, inexpensive and readily available biopolymer found in nature. It is contained at very high levels in cotton (~94%) and wood (~50%), which in turn are the major sources for cellulose products (paper, textiles, cosmetics, hygiene products, etc.) [1]. Traditionally, cellulose comes from vegetal resources and their waste.

Composite or engineered wood products such as particleboard (PB), plywood, waferboard or oriented strand board (OSB), medium density fibreboard (MDF) and the like are widely used in furniture manufacturing to replace the more expensive and scarcer natural wood (solid wood). For the manufacturing of the aforementioned wood products, it is necessary to mix or coat wood, coming from various forest species, in the form of particles, fibres, veneers or flakes, with special gluing systems, comprising adhesives (thermosetting polymer resins) and several chemical additives. Heat and pressure are then applied to form the final polymer network and bind the wood elements together and form the final wood panel product. The application of the adhesive system on the specially prepared wood parts is carried out by spraying the gluing system in the form of a mist through suitable spraying nozzles (PB, OSB, MDF), or spreading the adhesive system on the veneers (plywood) using, for example, a roller-coater to adequately distribute it on the wood surface. In the framework of EXILVA EU -funded -project, the objective of participant R&D company CHIMAR was to develop a new technology for the synthesis or the reinforcement of polymeric wood adhesives, by using EXILVA Micro-Fibrillated Cellulose (MFC), a product developed by Borregaard, based on an innovative technology for its production at commercial scale.

Micro-Fibrillated Cellulose (MFC) is characterized by its extended surface area with an expanded number of functional hydroxyl (-OH) groups. These characteristics are common for all MFC products. Moreover, MFC possesses several interesting properties, such as very high aspect ratios of the fibrillated fibres [2]. A number of reviews have shown mechanical improvement offered by MFC [3]. Therefore, it has a great potential to reinforce synthetic, petroleum-based resins that are already used in the market. In addition, positive effects of nanocellulose addition to amino-plastic adhesives in the bonding of particleboard and oriented strand panels have been found [4]. Particleboard panels have been manufactured using CNF (Cellulose Nano-Fibres, similar material to MFC) as a sole binder and met the industry requirements in terms of mechanical properties [5]. Another study revealed that MFC can be utilized as a rheology modifier, giving a more viscous urea-formaldehyde (UF) resin compared to the unmodified resin, in the manufacture of particleboard panels [6]. In the same study, a higher fraction of adhesive was proven to be available for bond-line formation and a larger part of the wooden particles was covered with adhesive, when MFC was used as a reinforcing agent of UF resin. Changed adhesive distribution together with improved adhesive toughness are proposed to contribute to improved board strength [6]. Nanocellulose reinforced UF resins have displayed improvement of storage modulus values and the wood composites manufactured with these reinforced UF resins showed enhanced performance [7]. A study on the effect of the length and content of cellulose fibers in phenol-formaldehyde (PF) resin showed an improvement in the mechanical properties of the wood composites manufactured with this resin, compared to the reference composites, manufactured with the unmodified PF resin [8].

In the framework of the present study, MFC was used in combination with currently used petrochemical adhesives, i.e., amino resins like urea-formaldehyde (UF) and melamine-urea-formaldehyde (MUF), and phenolic resins like phenol-formaldehyde (PF). The MFC was introduced either during the polycondensation stage of the resin synthesis or as an additive admixed together with the already prepared resin and other chemicals in a glue mixture, which eventually was sprayed onto wood particles or spread onto wood veneers, depending on the panel type. Three types of panels were manufactured at CHIMAR pilot plant: particleboard (PB), waferboard and plywood panels.

## 2. Materials and Methods

### 2.1. Introduction of MFC

**Figure 1:**
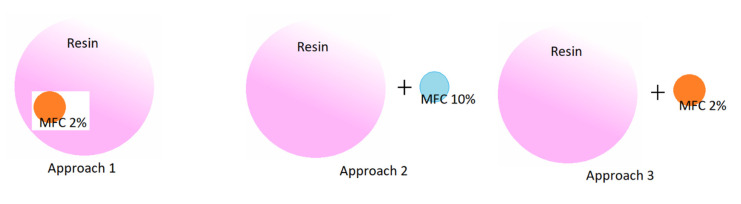
Approaches of introduction of MFC into adhesive systems.

Three different approaches to introduce MFC into adhesive systems were followed (Figure 1):

Approach 1: Introduction of MFC 2% suspension in the resin as a raw material, during its synthesis.

Approach 2: Admixing MFC 10% paste with the resin after its synthesis (addition level: 0.25–1.0% *w*/*w*, dry/dry resin).

Approach 3: Admixing MFC 2% suspension with the resin after its synthesis (addition level: 0.01–0.03% *w*/*w*, dry/dry resin).

Each approach was specially chosen for each adhesive type and the respective application (UF/MUF for particleboard panels, MUF for waferboard panels, PF for plywood panels), according to the best manufacturing practices of the wood-based panel sector.

The scope of following this path was to investigate whether MFC reacts chemically or physically with the resin as well as to evaluate its contribution to the improvement of the performance of the produced boards. It was assumed that when added during the resin synthesis (Approach 1) and under specific conditions (temperature, pH, etc.), MFC would participate in the polymer network. On the other side, when added into the resin system, after the synthesis of the resin (Approach 2, Approach 3), it would modify the physical properties of the resin, i.e., its rheology. All these different effects were investigated at CHIMAR R&D premises.

### 2.2. Materials

Formaldehyde 37% was supplied by PanReac (Barcelona, Spain). Phenol 90% was supplied by Merck (Darmstadt, Germany). The following industrial-grade reagents were supplied by Elton Group S.A. (Thessaloniki, Greece): urea, melamine, formic acid (80%), sodium hydroxide (50%). Ammonium sulphate was purchased by New Trade Fertilizers (Athens, Greece). EXILVA MFC, P-series, at concentrations of 2% (L-grade) and 10% (V-grade) were supplied by Borregaard (Sarpsborg, Norway). Wheat flour was also used as a filler in the production of plywood panels.

UF and MUF resins were applied in the production of particleboard panels, MUF resin was applied in the production of waferboard panels and PF resin was used for the production of plywood panels. All resins were synthesised at the laboratory of CHIMAR.

Ammonium sulphate, in the form of 40% aqueous solution, was used as hardener for the UF and MUF resins during the manufacture of particleboard and waferboard panels. For the production of plywood panels with 3 plies, the phenolic resin was mixed with wheat flour and water, in order to obtain the appropriate viscosity to be spread uniformly onto the wood veneers.

### 2.3. Synthesis of UF, MUF and PF Resins

The synthesis of UF resins was carried out according to a two-step process, comprising the methylolation and condensation stages. MFC 2% suspension was added at the beginning of the synthesis process, together with the rest of the raw materials (Approach 1). The methylolation step was realized by the addition of formaldehyde to urea to give the so-called methylolureas, followed by the addition of sodium hydroxide to adjust the pH to an alkaline value. The condensation reaction, which followed, was done at elevated temperature, in the acid condensation stage, using formic acid to reduce the pH. When the required viscosity was reached, the pH was adjusted again back to the alkaline region. At the end, a final portion of urea was added in order to reach a final F:U molar ratio in the range of 1.0–1.1. A reference UF resin was synthesised in parallel to the MFC-modified UF resin. The solid content of the resins was in the range of 64–66%. The MFC 2% suspension was added at a level of 0.22% *w*/*w* dry MFC on liquid resin. All other settings of the resin formulation were kept the same as those of the reference resin.

MUF resins were synthesised in a similar process to the UF resin process, including melamine as well as urea during the methylolation step. A reference MUF resin was synthesised in parallel to the MFC-modified MUF resin. The final F:(U + M) molar ratio was in the range of 1.0–1.1. The melamine content was within the range of 15–30%, depending on the application (particleboard or waferboard). The solid content of the resins was in the range of 64–66%. For Approach 1, MFC 2% suspension was added in the resin during its synthesis, at a level of 0.32% *w*/*w* dry MFC on liquid resin. All other settings of the resin formulations were kept the same as in the reference resin.

The synthesis of PF resins was realized according to the resole synthesis process comprising the hydroxymethylation and condensation stages. Formaldehyde was added to phenol, using sodium hydroxide as basic catalyst, for the hydroxymethylation to occur. The condensation reaction took place afterwards, at increased temperature. When the desired viscosity was reached, the resin was cooled down to room temperature and stored at 5 °C, as suggested for this type of resin. A reference PF resin was synthesised in parallel to the MFC-modified PF resin. The final F:P molar ratio was in the range of 2.0–2.2 and the resin solid content in the range of 40–45%. For Approach 1, MFC 2% suspension was added in the resin during its synthesis, at a level of 0.22% *w*/*w* dry MFC on liquid resin. All other settings of the resin formulation were kept the same as in the reference resin.

All resins were delivered in liquid form.

### 2.4. Resin Characterization

The properties of the produced resins were determined based on standard lab analysis methods. All the produced UF and MUF (amino) resins were characterized for their solid content, pH value, viscosity, gelation time, specific gravity and water tolerance. The solid content was determined by drying 2 g of resin in an oven at 120 °C for 2 h. The pH value of the resins was measured using a digital GLP21 pH-meter from CRISON (Barcelona, Spain) with a single Hamilton glass electrode attached. The viscosity was measured at 25 °C using a Brookfield rotational viscometer, with a small sample adapter (SC4-45Y), which requires a sample chamber SC4-13R and a spindle SC4-18. The gelation time of the resins was determined by measuring the time needed for gelation in boiling water after addition of 3.5% ammonium sulphate hardener (*w*/*w* dry/dry resin). For the determination of the water tolerance of the resins at 25 °C, a 10 mL sample of resin was added in a volumetric cylinder and the quantity of water needed until the sample started to coagulate was measured. The specific gravity was determined using suitable glass hydrometers.

Additionally, Differential Scanning Calorimetry (DSC) analysis was performed for specific experiments with UF resins, which were used for particleboard production, to evaluate the curing behavior of the UF resins with and without the addition of MFC. The testing was done in a Shimadzu DSC-50 device, using Aluminum High Pressure Cells (222-01701-91). In all measurements, the resin was catalysed with 3,5% *w*/*w* ammonium sulphate (in the form of 40% *w*/*w* aqueous solution) and all formulations were adjusted to the same solid content, by addition of water where needed. Test samples of 14–16 mg for each variable were placed in the aluminum cells, sealed and measured immediately. All samples were heated up to 200 °C at a heating rate of 10 °C/min under nitrogen atmosphere. For each test sample, the peak temperature, the onset temperature and the heat release were recorded.

For the PF resins’ characterization, the solid content, pH value, viscosity, gelation time and alkalinity were determined. The pH value, the viscosity, the gelation time and the solid content measurements were in accordance with the practice followed for the UF and MUF resins. The alkalinity measurement is a titration with HCl acid, to calculate the remaining NaOH in the resin.

### 2.5. Panel Manufacturing Process

Three types of panels were manufactured at CHIMAR pilot plant: particleboard (PB), waferboard and plywood panels. The panel production process followed the industrial manufacturing practice. The mechanical and wet properties of the boards produced were determined in accordance with the European standards (EN) in force, i.e., density (EN 323), tensile strength/Internal Bond (IB) (EN 310), bending strength/Modulus Of Rupture (MOR)/Modulus Of Elasticity (MOE) (EN 319), Thickness Swelling (TS) (EN 317), as well as the formaldehyde emission potential, were measured by determination of the Formaldehyde content according to the Perforator Method (International Standard ISO 12460-5). The properties of the panels should satisfy the specifications as stated in product standards EN 312 (PB), EN-300 (Waferboard/OSB) and EN-314.02 (plywood).

#### 2.5.1. Particleboard

Particleboard panels were manufactured at CHIMAR wood-based panels pilot production plant, according to industrial practice. The gluing mixture, consisting of resin, hardener and water, was sprayed on the wood particles, consisting mainly of a mixture of pine with 20%–30% poplar wood, and having a moisture content of ca. 3%. The particles were blended with the gluing mixture in a Lödige FM130D blender equipped with a suitable spraying system attached on the top side, for about 3 min. The resinated wood particles were then formed in a mattress (mat) with dimensions of 44 × 44 cm. The mat was manually pre-pressed, and it was then led to the hydraulic press and pressed at high temperature for a sufficient time to achieve the cross-linking and total curing of the resin. The pressing temperature and time were defined by previous experience with similar experiments and were the same for all particleboard panels produced. The target density of the boards was 650 kg/m^3^ and the target thickness was 15 mm. After they were given time to cool down, the produced boards were sanded and proper specimens were taken from them, for testing the board mechanical and wet properties, as well as their formaldehyde content, according to the perforator method (ISO 12460-5). Each resin system formulation was applied in duplicates of panels.

#### 2.5.2. Waferboard/Randomly-Oriented Strand Board

The production of waferboard panels was carried out following a very similar procedure to that of the particleboard, but using different blending and spaying devices. A cement mixer was used as a blending device, in order to avoid breaking down the wood strands. The glue mixture was sprayed through a high-pressure handheld spraying gun. The mat forming and the pressing process were the same as in the case of particleboards production. The pressing temperature and time were defined by previous experience with similar experiments and were the same for all the panels produced. The target density of the boards was 600 kg/m^3^ and the target thickness was 15 mm. Each formulation consisted of one panel.

#### 2.5.3. Plywood

For the plywood manufacture, a glue mixture consisting of resin, wheat flour and water was prepared and spread on the wood veneers (50 × 50 cm × 1.5 mm), with the assistance of a spatula and a roller. The wood species that was used for the present study was beech. The glued veneers were stacked and pre-pressed. Afterwards, they were pressed in the hydraulic press at high temperature for a sufficient time to totally cure the resin. The pressing temperature and time were defined by previous experience with similar experiments and were common for all plywood panels. After having been cooled down, the produced panels were cut in specimens and their mechanical properties were determined. Each resin system formulation was applied in duplicates of panels.

## 3. Results

During the experimental part, a lot of different cases were investigated, concerning the MFC concentration, the MFC addition levels (dry/liquid resin), the resin loading levels, the resin and panel type, etc., and they are all presented in the following sections, divided according to the panel type.

### 3.1. Particleboard Production

As mentioned earlier, the resins that were utilized for the particleboard manufacture were of UF and MUF type. Table 1 below presents the analysis results from the characterization of a conventional and an MFC-reinforced resin of both types (the latter synthesized according to Approach 1).

The analysis results indicate that there is no significant difference between the properties of the reference UF resin and the ones of the MFC-reinforced UF resin. When comparing the reference MUF resin with the MFC-reinforced one, there is a notably shorter gel time (higher reactivity) of the reference resin, which could contribute to its better performance in particleboard mechanical properties against the MFC reinforced MUF resin, as presented in the next set of experiments.

#### 3.1.1. MFC 2% Suspension Either Added as Raw Material of UF or MUF Resin during Synthesis or Admixed with the Ready UF or MUF Resin (Approach 1 vs. Approach 3)

The results obtained from the analysis of the properties of the panels produced during experiment 3.1.1 are presented in Table 2.

It is clear that for both types of resin, UF and MUF, when Approach 1 is followed and MFC 2% is introduced in the resin during its synthesis, the mechanical properties and especially the tensile strength (Internal Bond) are impaired. When Approach 3 is followed and MFC 2% is admixed with the ready resin, the internal bond is improved in both UF and MUF cases. The water resistance of the boards is not significantly influenced in either case. The bending properties (modulus of rupture and modulus of elasticity) follow a trend similar to the tensile strength, which is, however, not considered to be significant. The formaldehyde content of the board seems to be reduced by Approach 1 in the UF resin, while in the MUF case, the formaldehyde content is increased by the addition of MFC. On the other side, when Approach 3 is followed in the UF resin case, the formaldehyde content is not affected, while in the MUF case, it is further increased by the addition of MFC.

#### 3.1.2. MFC 10% Paste at Increasing Addition Levels, Keeping the Resin Loading Level Stable. MFC Admixed with UF Resin (Approach 2)

The results obtained from the analysis of the properties of the panels produced during experiment 3.1.2 are presented in Table 3.

When MFC 10% is admixed with the UF resin at elevated levels of addition, it proportionally improves the mechanical properties, with more significant differences appearing on the tensile strength results. Water resistance of the boards does not seem to be importantly affected. The fluctuation of the results obtained for the Formaldehyde content is within the accuracy range, so no conclusions can be drawn.

#### 3.1.3. MFC 10% Paste at Increasing Addition Levels, Decreasing the Resin Loading Level. MFC Admixed with UF Resin (Approach 2)

The results obtained from the analysis of the properties of the panels produced during experiment 3.1.3 are presented in Table 4.

This experiment proves that when MFC is admixed with the resin up to a specific level (0.25% dry/liquid resin), it allows the reduction in the resin loading level, since all board properties are equal to or even better than in the reference formulation (comparison between the two first formulations). This tendency disappears by further reduction in resin loading, even with an increase in the addition levels of MFC. Beyond that point, it seems that the influence of the reduction in the petrochemical resin is more significant than the introduction of MFC at increasing levels, and this becomes evident from the results of all properties.

#### 3.1.4. MFC 2% Suspension at Increasing Addition Levels, Keeping the Resin Level Stable. MFC Admixed with UF Resin (Approach 3), Average Property Values, Standard Deviation (in Brackets)

The results obtained from the analysis of the properties of the panels produced during experiment 3.1.4 are presented in Table 5.

Admixing MFC 2% suspension with the ready resin at low levels 0.01–0.03% dry/liquid resin (Approach 3) shows a significant advantage of increased levels of MFC to the mechanical properties and water resistance of the produced boards. Formaldehyde content was gradually decreased when increasing the MFC levels, which is a positive effect too.

#### 3.1.5. MFC 2% Suspension at Increasing Addition Levels, Decreasing the Resin Loading Level. MFC Admixed with UF Resin (Approach 3), Average Property Values, Standard Deviation (in Brackets)

The results obtained from the analysis of the properties of the panels produced during experiment 3.1.5 are presented in Table 6.

When MFC 2% suspension is admixed with the ready resin, at gradually increasing but low dry/liquid resin levels (Approach 3) with parallel reduction in the resin level, the boards’ tensile strength is improved even at a 17% decrease in the resin level. The water resistance and bending strength of the boards are slightly impaired, but the fluctuation of the results is within the accuracy range, so no significant differences are noted. Formaldehyde content is, respectively, reduced.

The slight differences observed in the results for the same formulations among the various experiments, derive from the variability of the process and possibly from different prevailing environmental conditions.

### 3.2. Waferboard Production

For the manufacture of waferboard panels, MUF resin type was used. The results from the analysis of one resin sample of this type are presented in Table 7:

#### 3.2.1. MFC 10% Paste Admixed with MUF Resin at Increasing Addition Levels, Keeping the Resin Level Stable (Approach 2), Average Property Values, Standard Deviation (in Brackets)

The results obtained from the analysis of the properties of the panels produced during experiment 3.2.1 are presented in Table 8.

The analysis of the produced waferboard panels following Approach 2 (admixing MFC 10% paste with the ready resin) indicated that only adding 1.00% dry MFC on liquid MUF resin significantly improves the Internal Bond and slightly improves the Modulus of Rupture/Modulus of Elasticity and water resistance of the produced boards. The latter effect is shown by the decrease in thickness swelling. The formaldehyde content was not affected. However, the higher the MFC level, the more viscous the glue mixture, almost reaching the limit of being sprayable when that level of MFC is mixed with the resin.

#### 3.2.2. MFC 10% Paste Admixed with MUF Resin at Increasing Addition Levels, Decreasing the Resin Level (Approach 2), Average Property Values, Standard Deviation (in brackets)

The results obtained from the analysis of the properties of the panels produced during experiment 3.2.2 are presented in Table 9.

Up to a certain point, 11.3% MUF/0.25% MFC, admixing MFC 10% paste with the resin (Approach 2), even at reduced resin loading level, could be proven advantageous, especially concerning mechanical properties. The water resistance (thickness swelling and water absorption) is also positively affected by the addition of MFC. Beyond that point, all the properties start to be impaired, probably because the influence of the reduction in the petrochemical resin is more significant than the introduction of MFC. The strong hydrophilic character of the MFC, which is due to the high amount of -OH groups on its surface, possibly leads to higher water adsorption, reducing the water resistance of the produced boards. The fluctuation of the results obtained for the formaldehyde content is within the accuracy range, so no conclusions can be drawn.

The slight differences observed in the results for the same formulations among the various experiments derive from the variability of the process and possibly from different prevailing environmental conditions.

### 3.3. Plywood Production

For the manufacture of plywood panels, PF resins were synthesized and the results from the analysis of a reference PF resin and of an MFC-reinforced one (Approach 1), used during this series of experiments, are presented in Table 10.

A comparison between the reference PF resin and the MFC-reinforced one concerning their analysis results indicates a shorter gel time for the reference PF, and thus a higher reactivity, which, however, could cause premature resin curing reaction. Moreover, the viscosity of the MFC-reinforced PF is higher, which makes sense, since MFC can operate as a thickener and thus it could, as well, prevent the over-penetration of the glue into the wood substrate.

#### 3.3.1. MFC 2% Suspension Added as Raw Material in PF Resin during its Synthesis (Approach 1), Average Property Values, Standard Deviation (in Brackets)

The results obtained from the analysis of the properties of the panels produced during experiment 3.3.1 are presented in Table 11.

When MFC 2% suspension was added in the PF resin during its synthesis (Approach 1), it helped to improve the shear strength and achieve equal wood failure as compared to the reference plywood panels. Both characteristics mentioned above, i.e., lower resin reactivity and higher viscosity, could have contributed to the improved shear strength of the panels with the MFC-reinforced formulation, apart from the fact that MFC participates in the formulation as well.

#### 3.3.2. MFC 10% Paste Evaluated as Rheology Modifier (Thickener), Replacing Wheat Flour, Admixed with the Ready PF Resin (Approach 2), Average Property Values, Standard Deviation (in Brackets)

The results obtained from the analysis of the properties of the panels produced during experiment 3.3.2 are presented in Table 12.

In this case, the addition level of MFC 10% paste was chosen by preparing a glue mixture with similar viscosity to that of the reference glue mixture containing flour. It was observed that the MFC-based formulation was spread more smoothly and evenly than the reference and it prevented early penetration of the glue mixture in the wood substrate, which translates to a rheology improvement of the glue mixture. The panel properties of the two formulations were similar.

#### 3.3.3. MFC 10% Paste Admixed with PF Resin at Elevated Addition Levels, Keeping the Resin Loading Level Stable (Approach 2), Average Property Values, Standard Deviation (in Brackets)

The results obtained from the analysis of the properties of the panels produced during experiment 3.3.3 are presented in Table 13.

There was no statistically significant positive effect on the shear strength of plywood panels when the MFC addition levels according to Approach 2 were gradually increased. It was shown that an addition level from 0.23 to 0.46% of dry MFC/liquid resin is enough to slightly improve the % wood failure.

The slight differences observed in the results for the same formulations among the various experiments derive from the variability of the process and possibly from different prevailing environmental conditions.

In total, MFC seems to modify the rheology of the adhesive systems where it is introduced, by increasing their viscosity, while allowing a homogeneous distribution of the resin mixture when applied on the wood. This phenomenon could be attributed to the non-Newtonian character rendered to the resin mixture by MFC, which brings a shear thinning behaviour to it. Such a characteristic is important in the manufacturing process of wood-based panels, since the glue mixture is sprayed on the wood-particles/wood-strands under very high pressure, through spraying nozzles, which create a very fine mist of the mixture. When MFC participates in the mixture, the shear thinning effect facilitates the easy spray of the resin mixture, which is then evenly distributed on the wood particles. The small resin droplets regain their viscosity afterwards, and remain on the wood surface, thus avoiding the resin loss due to absorption of the resin mixture from the wood substrate, which happens at a high extent in the case of the reference, without MFC, formulations. The avoidance of resin loss leads to optimum adhesion performance and improved product properties. In this study, this effect was evident in the case of particleboard and RSB (waferboard), when MFC was admixed with the resin during the preparation of the glue mixture, either at high addition levels (MFC 10% paste/Approach 2) or at low addition levels (MFC 2% suspension/Approach 3).

### 3.4. Differential Scanning Calorimetry (DSC)

The potential effect of MFC addition on the curing behaviour of the UF adhesive, in some of the experiments described above, was evaluated by Differential Scanning Calorimetry (DSC). In particular, the DSC analysis was performed for the UF adhesive systems for experiments 3.1.1, 3.1.2 and 3.1.4, in order to evaluate the MFC influence when used in combination with UF resins via all three approaches in particleboard production. Since UF resins are thermosetting polymers, their curing reaction is an exothermal one, as depicted by the peaks of the DSC plots, which are presented henceforward. The DSC analysis results are also presented in the tables below:

Experiment 3.1.1. MFC 2% Suspension in UF Resin during Synthesis or Admixed with the Ready UF Resin (Approach 1 vs. Approach 3)

**Figure 2:**
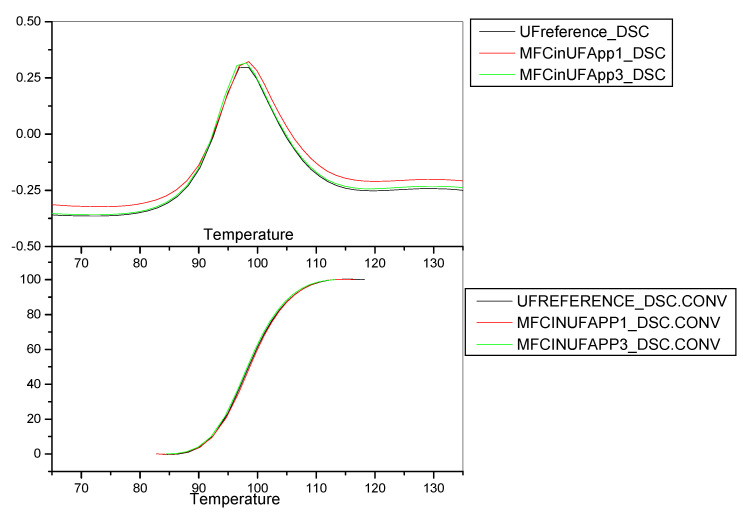
DSC curves, Experiment 3.1.1.

The results obtained from the DSC analysis of the resin systems used during experiment 3.1.1 are presented in Table 14 and the corresponding thermograms are depicted in Figure 2.

The results obtained from the DSC analysis of the first experiment suggest that the reactivity of the UF resins including MFC (either during the resin synthesis or admixed with the resin) was impaired, since higher temperature is necessary for the curing process to take place, according to the respective peak temperatures obtained. It was also observed that the MFC-reinforced UF resin (Approach 1) cures at relatively higher temperature than the UF resin where MFC was admixed (Approach 3). These results are in agreement with the particleboard analysis results, where Approach 3 was proven to be advantageous against Approach 1.

Experiment 3.1.2. MFC 10% Paste at Increasing Addition Levels, Keeping the Resin Loading Level Stable. MFC Admixed with UF Resin (Approach 2)

**Figure 3:**
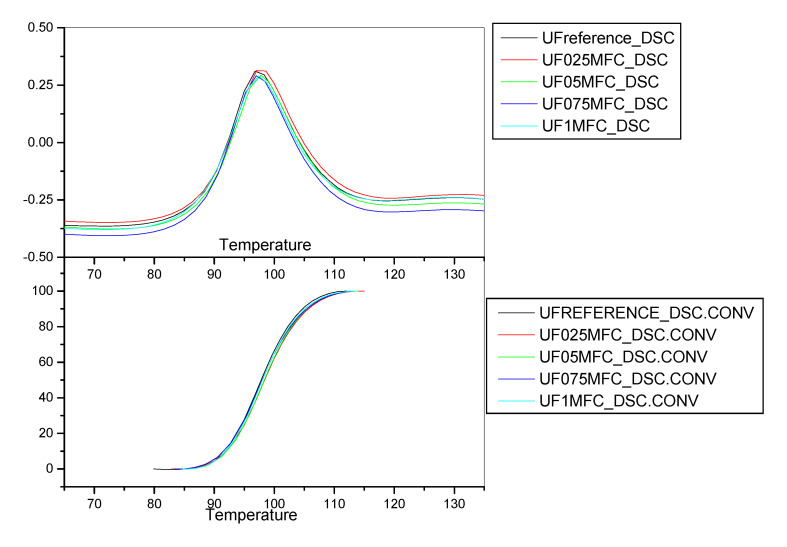
DSC curves, Experiment 3.1.2.

The results obtained from the DSC analysis of the resin systems used during experiment 3.1.2 are presented in Table 15 and the corresponding thermograms are depicted in Figure 3.

The results obtained in this case suggest that the reference UF resin formulation is more reactive than the formulations where MFC was added. This is not in accordance with the particleboard analysis results. It should be noted, however, that in board production, the curing behaviour of the adhesive system is usually affected by the participation of the wood substrate, due to the pH value and the pH buffering capacity of wood, and this may the reason for the difference between the DSC and the particleboard production results. Furthermore, the improved behaviour of the MFC-containing formulations may be attributed to the rheology effect of the MFC on the resin mixtures during the particleboard manufacturing process, as explained previously. On the other side, the DSC results show that the onset temperature declines with the increase in MFC level in the system, which indicates an increase in reactivity, a result conforming with the particleboard analysis results.

Experiment 3.1.4. MFC 2% Suspension at Increasing Addition Levels, Keeping the Resin Level Stable. MFC Admixed with UF Resin (Approach 3)

**Figure 4:**
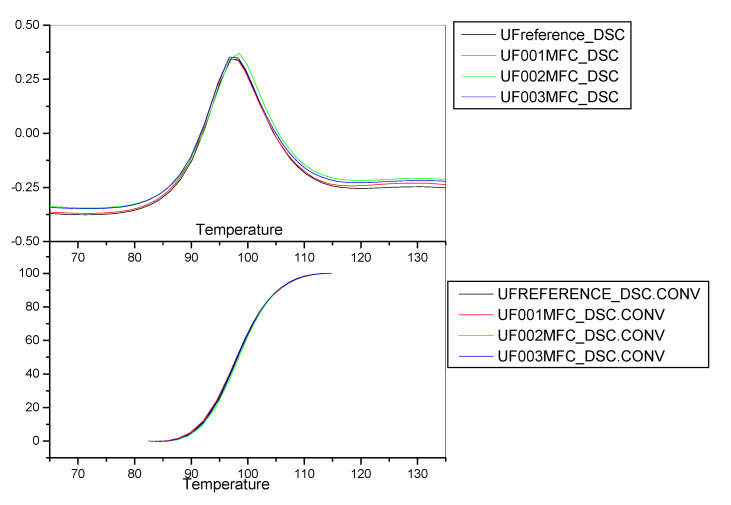
DSC curves, Experiment 3.1.4.

The results obtained from the DSC analysis of the resin systems used during experiment 3.1.4 are presented in Table 16 and the corresponding thermograms are depicted in Figure 4.

The results obtained from this DSC analysis suggest that the most reactive formulation is that with the lowest MFC addition level, followed by the reference UF formulation, especially when observing the onset temperatures. In addition, the highest temperature necessary for the curing reaction to take place was obtained by the analysis of the formulation with the highest MFC addition level. However, the particleboard results indicated that the higher the MFC addition level, the better the board performance. It is hence deduced that here, again, the wood substrate interaction with the resin in combination with the MFC addition may have positively influenced the curing process of the adhesive system and also the board performance.

## 4. Discussion and Conclusions

The conclusions drawn from the entire experimental process are the following:

Particleboard

- The introduction of MFC 2% suspension in the UF and MUF resins during their synthesis (Approach 1), at a range of 0.22% and 0.32% (dry MFC/liquid resin), respectively, deteriorates the performance of particleboard panels, especially the internal bond.
- When MFC 2% suspension is mixed with the ready UF and MUF resins in the glue mixture (Approach 3), at the same range of 0.22% and 0.32% (dry MFC/liquid resin) as in Approach 1, respectively, the performance of particleboard panels improves, especially the internal bond. The other panel properties are not significantly affected.
- Increasing the addition levels of MFC 10% paste in UF resin (Approach 2), at a range of 0.25%–1.00% (dry MFC/liquid resin), improves the tensile strength (internal bond) of the produced particleboards. The other panel properties are not significantly affected.
- The addition of MFC 10% paste in UF resin (Approach 2), up to a level of 0.25% (dry MFC/liquid resin), allows the reduction in UF resin consumption in particleboard panels, by maintaining or even improving the board performance.
- Increasing addition levels of MFC 2% suspension in UF resin (Approach 3), at a range of 0.01%–0.03% (dry MFC/liquid resin), clearly improves the performance of the produced boards.
- The addition of MFC 2% suspension in UF resin (Approach 3), within a range of 0.01%–0.03% (dry MFC/liquid resin), allows the reduction in UF resin consumption in particleboard panels, by improving the internal bond and reducing the formaldehyde content of the boards but slightly impairing the other panel properties.
- MFC acts as a rheology modifier of the resin mixture, improving its distribution on the wood particles, enabling it to cover a larger surface of wood, and preventing its penetration into the wood substrate.

Waferboard

- Increasing the addition levels of MFC 10% paste in MUF resin (Approach 2), within a range of 0.25%–1.00% (dry MFC/liquid resin), improves the mechanical properties of waferboard panels.
- The addition of MFC 10% paste in MUF resin (Approach 2), up to a level of 0.50% (dry MFC/liquid resin), allows the reduction in MUF resin consumption in waferboard panels, maintaining the board properties.
- Similarly to the particleboard case, MFC can act as a rheology modifier of the resin mixture of the RSB application as well, improving its distribution on the wood strands, enabling it to cover a larger surface of wood and preventing its penetration into the wood substrate.

Plywood

- The introduction of MFC 2% suspension in the PF resin during its synthesis (Approach 1), at an addition level of 0.22%, improves the performance of plywood panels.
- Increasing the addition levels of MFC 10% paste in PF resin (Approach 2), within a range of 0.23%–0.68% (dry MFC/liquid resin), did not offer any significant improvement to the board properties. Nevertheless, MFC can be added as a thickener in the glue mixture of the PF resin for plywood production, substituting the conventionally-applied wheat flour, while maintaining the panel performance.

The conclusions discussed above can be better illustrated in Table 17 below:

Table 17 indicates that the addition of MFC 2% suspension in UF resin during particleboard production, at addition levels of 0.01–0.03% (dry MFC/liquid resin), following Approach 3, is the most promising path to follow.

Summarising, the use of MFC in adhesive systems for wood-based panels’ production has shown positive results at laboratory scale. When introduced at low addition levels as an additive in the glue mixture of the resin, it improves board performance and allows the reduction in petrochemical substances, such as, for instance, the resin itself.

The findings of this research work pave the way for the development and adoption of adhesive formulations with lower CO_2_ footprint, rendering a more environmentally friendly character to the final wood-based panel products.

## Figures and Tables

**Table 1:** Urea-Formaldehyde (UF) and Melamine-Urea-Formaldehyde (MUF) resins analysis results, Particleboard.

Resin Type	UF Reference	MFC-Reinforced UF	MUF Reference	MFC-Reinforced MUF
% MFC, dry/liquid resin	0.00	0.22	0.00	0.32
Solids (%)	65.8	67.0	63.6	63.6
pH	8.5	7.9	9.2	9.6
Viscosity (cP)	315	370	195	450
Gel time (s)	60	56	73	95
Water tolerance (mL:mL)	1/1.4	1/0.6	1/0.5	1/0.6
Specific gravity	1.279	1.295	1.265	1.233

**Table 2:** Particleboard panels analysis, MFC 2% suspension, Approach 1 vs. Approach 3, Average property values, Standard deviation ^1^ (in brackets).

Formulation	UF Reference	MFC-Reinforced UF (Approach 1)	UF + 0.22% MFC (Approach 3)	MUF Reference	MFC-Reinforced MUF (Approach 1)	MUF + 0.32% MFC (Approach 3)
% Binder, dry/dry wood	9.0	9.0	9.0	9.0	9.0	9.0
% MFC, dry/liquid resin	0.00	0.22	0.22	0.00	0.32	0.32
Internal bond (MPa)	0.58 (0.04)	0.52 (0.06)	0.63 (0.03)	0.90 (0.09)	0.82 (0.10)	0.95 (0.06)
24 h Thickness Swelling (%)	54.9 (4.4)	54.1 (4.1)	62.6 (4.0)	27.2 (1.4)	26.9 (0.7)	29.3 (1.7)
24 h Water Absorption (%)	100 (1.6)	96 (1.0)	99 (1.0)	72 (2.3)	76 (1.8)	75 (2.2)
Modulus of Rupture (MPa)	13.7	13.5	13.9	17.3	15.8	16.2
Modulus of Elasticity (MPa)	2641	2568	2418	2677	2627	2335
Formaldehyde content (mg/100 g ODB ^2^)	6.7	5.6	6.7	5.4	6.7	7.4

^1^ Standard deviation was added on properties where adequate population data existed. ^2^ Oven dried board.

**Table 3:** Particleboard panels analysis, MFC 10% increasing levels, resin stable, Approach 2, Average property values, Standard deviation in brackets).

Formulation	UF Reference	UF + 0.25% MFC	UF + 0.5% MFC	UF + 0.75% MFC	UF + 1.0% MFC
% Binder, dry/dry wood	7.0	7.0	7.0	7.0	7.0
% MFC, dry/liquid resin	0.00	0.25	0.50	0.75	1.00
Internal bond (MPa)	0.30 (0.04)	0.33 (0.06)	0.36 (0.03)	0.39 (0.05)	0.45 (0.04)
24 h Thickness Swelling (%)	45.3 (2.5)	45.5 (3.1)	42.9 (2.1)	42.8 (2.6)	43.6 (2.1)
24 h Water Absorption (%)	97 (1.9)	96 (1.8)	95 (1.6)	99 (2.1)	99 (2.2)
Modulus of Rupture (MPa)	13.4	13.4	13.5	13.6	13.9
Modulus of Elasticity (MPa)	2389	2402	2412	2445	2455
Formaldehyde content (mg/100 g ODB)	8.7	9.2	9.1	9.0	8.5

**Table 4:** Particleboard panels analysis, MFC 10% increasing levels, resin decreasing, Approach 2, Average property values, Standard deviation (in brackets).

Formulation	7.0% UF Reference	6.4% UF + 0.25% MFC	5.8% UF + 0.5% MFC	5.4% UF + 0.75% MFC	5.0% UF + 1.0% MFC	4.7% UF + 1.0% MFC
% Binder, dry/dry wood	7.0	6.4	5.8	5.4	5.0	4.7
% MFC, dry/liquid resin	0.00	0.25	0.50	0.75	1.00	1.00
Internal bond (MPa)	0.36 (0.02)	0.36 (0.02)	0.31 (0.03)	0.29 (0.02)	0.26 (0.02)	0.24 (0.02)
24 h Thickness Swelling (%)	54.1 (1.3)	51.3 (3.8)	59.1 (2.5)	64.8 (2.6)	65.9 (3.2)	72.3 (2.3)
24 h Water Absorption (%)	126 (2.4)	120 (2.3)	127 (4.3)	138 (1.5)	139 (2.8)	146 (2.9)
Modulus of Rupture (MPa)	9.6	11.7	10.5	8.9	8.3	7.3
Modulus of Elasticity (MPa)	1842	1994	1879	1695	1713	1443
Formaldehyde content (mg/100 g ODB)	8.0	7.4	7.3	8.2	8.3	8.3

**Table 5:** Particleboard panels analysis, MFC 2% increasing levels, resin stable, Approach 3.

Formulation	UF Reference	UF + 0.01% MFC	UF + 0.02% MFC	UF + 0.03% MFC
% Binder, dry/dry wood	9.0	9.0	9.0	9.0
% MFC, dry/liquid resin	0.00	0.01	0.02	0.03
Internal bond (MPa)	0.42 (0.02)	0.59 (0.02)	0.66 (0.03)	0.67 (0.06)
24 h Thickness Swelling (%)	44.2 (2.6)	41.4 (1.5)	39.4 (0.9)	40.0 (2.3)
24 h Water Absorption (%)	92 (3.8)	87 (4.2)	86 (4.1)	83 (5.4)
Modulus of Rupture (MPa)	10.1	11.2	11.7	11.6
Modulus of Elasticity (MPa)	2146	2198	2273	2275
Formaldehyde content (mg/100 g ODB)	7.5	7.0	6.2	6.2

**Table 6:** Particleboard panels analysis, MFC 2% increasing levels, resin decreasing, Approach 3.

Formulation	9.0% UF Reference	8.5% UF + 0.01% MFC	8.0% UF + 0.02% MFC	7.5% UF + 0.03% MFC
% Binder, dry/dry wood	9.0	8.5	8.0	7.5
% MFC, dry/liquid resin	0.00	0.01	0.02	0.03
Internal bond (MPa)	0.38 (0.03)	0.43 (0.03)	0.43 (0.05)	0.45 (0.04)
24 h Thickness Swelling (%)	58.0 (2.6)	59.0 (2.7)	58.6 (1.7)	61.6 (3.6)
24 h Water Absorption (%)	112 (3.1)	114 (3.3)	118 (2.2)	119 (3.4)
Modulus of Rupture (MPa)	9.7	9.9	9.5	8.8
Modulus of Elasticity (MPa)	2045	2095	2012	1917
Formaldehyde content (mg/100 g ODB)	10.5	9.2	8.6	8.0

**Table 7:** MUF resin analysis results, Waferboard.

Resin Type	MUF
Solids (%)	64.2
pH	9.3
Viscosity (cP)	360
Gel time (s)	66
Water tolerance (mL:mL)	1/0.5
Specific gravity	1.279

**Table 8:** Waferboard panels analysis, MFC 10% increasing levels, resin stable, Approach 2.

Formulation	MUF Reference	MUF + 0.25% MFC	MUF + 0.50% MFC	MUF + 0.75% MFC	MUF + 1.00% MFC
% Binder, dry/dry wood	12.0	12.0	12.0	12.0	12.0
% MFC, dry/liquid resin	0.00	0.25	0.50	0.75	1.00
Internal bond (MPa)	0.50 (0.06)	0.49 (0.08)	0.51 (0.10)	0.52 (0.03)	0.61 (0.06)
24 h Thickness Swelling (%)	22.1 (2.1)	24.5 (2.8)	22.4 (0.8)	20.8 (0.9)	19.8 (5.3)
24 h Water Absorption (%)	61 (2.2)	68 (3.3)	65 (7.4)	60 (7.2)	61 (6.3)
Modulus of Rupture (MPa)	21.4	20.8	23.1	22.1	24.1
Modulus of Elasticity (MPa)	3395	3257	3679	3820	3789
Formaldehyde content (mg/100 g ODB)	7.4	6.8	7.4	7.8	7.5

**Table 9:** Waferboard panels analysis, MFC 10% increasing levels, resin decreasing, Approach 2.

Formulation	12% MUF Reference	11.3% MUF + 0.25% MFC	10.5% MUF + 0.50% MFC	9.8% MUF + 0.75% MFC	9.0% MUF + 1.00% MFC
% Binder, dry/dry wood	12.0	11.3	10.5	9.8	9.0
% MFC, dry/liquid resin	0.00	0.25	0.50	0.75	1.00
Internal bond (MPa)	0.58 (0.13)	0.65 (0.12)	0.59 (0.09)	0.54 (0.11)	0.57 (0.08)
24 h Thickness Swelling (%)	21.1 (2.1)	19.9 (1.1)	22.8 (2.5)	25.4 (2.5)	29.8 (2.7)
24 h Water Absorption (%)	66 (4.4)	63 (5.2)	65 (3.8)	83 (10.2)	78 (5.5)
Modulus of Rupture (MPa)	18.3	19.5	18.3	15.0	16.0
Modulus of Elasticity (MPa)	2923	3295	3069	2600	2711
Formaldehyde content (mg/100 g ODB)	7.3	7.1	7.0	6.7	7.0

**Table 10:** Phenol-Formaldehyde (PF) resin analysis results, Plywood.

Resin Type	PF Reference	MFC-Reinforced PF
% MFC, dry/liquid resin	0.00	0.22
Solids (%)	40.6	40.9
pH	11.8	11.6
Viscosity (cP)	360	430
Gel time (min)	17	26
Alkalinity (%)	6.8	6.6

**Table 11:** Plywood panels analysis, MFC 2%, Approach 1.

Formulation	PF Reference	MFC-Reinforced PF (Approach 1)
Binder, g/m^2^	58	58
% MFC, dry/liquid resin	0.00	0.22
Shear Strength, N/mm^2^	1.2 (0.49)	1.5 (0.41)
Wood failure, %	72 (16)	70 (25)

**Table 12:** Plywood panels analysis, MFC 10%, Approach 2.

Formulation	PF Reference	PF + 0.50% MFC (Approach 2)
Binder, g/m^2^	58	58
% MFC, dry/liquid resin	0.00	0.50
% Wheat flour, dry/liquid resin	12.0	0.0
% Water, liquid/liquid resin	8.0	0.0
Shear Strength, N/mm^2^	1.4 (0.56)	1.5 (0.48)
Wood failure, %	78 (17)	73 (19)

**Table 13:** Plywood panels analysis, MFC 10% increasing levels, Approach 2.

Formulation	PF Reference	PF + 0.23% MFC	PF + 0.46% MFC	PF + 0.68% MFC
Binder, g/m^2^	58	58	58	58
% MFC, dry/liquid resin	0.00	0.23	0.46	0.68
Shear Strength, N/mm^2^	1.5 (0.61)	1.3 (0.68)	1.5 (0.66)	1.5 (0.58)
Wood failure, %	72 (21)	82 (22)	80 (25)	76 (24)

**Table 14:** DSC analysis results, Experiment 3.1.1 (Approach 1 vs. Approach 3).

Sample	Peak (°C)	Onset (°C)	Heat (J/g)
UF Reference	96.95	88.41	45.22
MFC-Reinforced UF (Approach 1)	98.51	88.20	43.94
UF + 0.22% MFC (Approach 3)	98.10	88.15	47.26

**Table 15:** DSC analysis results, Experiment 3.1.2 (Approach 2).

Sample	Peak (°C)	Onset (°C)	Heat (J/g)
UF reference	96.81	86.76	46.41
UF + 0.25% MFC	97.04	88.36	45.12
UF + 0.50% MFC	97.76	87.28	45.00
UF + 0.75% MFC	96.98	86.95	49.02
UF + 1.00% MFC	97.38	86.83	47.49

**Table 16:** DSC analysis results, Experiment 3.1.4 (Approach 3).

Sample	Peak (°C)	Onset (°C)	Heat (J/g)
UF Reference	97.02	86.35	50.25
UF + 0.01% MFC	98.12	86.06	50.26
UF + 0.02% MFC	96.95	88.41	45.22
UF + 0.03% MFC	98.51	88.20	43.94

**Table 17:** Conclusions and general evaluation.

Application	Approach	MFC Addition Level, % Dry MFC/Liquid Resin	MFC Grade	Property	Outcome	Remarks
Particleboard	1	0.22–0.32%	2% suspension	Mechanical properties (Internal Bond, Modulus of Rupture, Modulus of Elasticity)	Negative	
				Wet properties (Thickness swelling, Water absorption)	Negative	
				Formaldehyde content	Positive	
	3	0.22–0.32%	2% suspension	Mechanical properties (Internal Bond, Modulus of Rupture, Modulus of Elasticity)	Positive	
				Wet properties (Thickness swelling, Water absorption)	Neutral	
				Formaldehyde content	Neutral	
	2	0.25–1.00%	10% paste	Mechanical properties (Internal Bond, Modulus of Rupture, Modulus of Elasticity)	Positive	Allows reduction in resin, maintaining board properties
				Wet properties (Thickness swelling, Water absorption)	Neutral	
				Formaldehyde content	Neutral	
	3	0.01–0.03%	2% suspension	Mechanical properties (Internal Bond, Modulus of Rupture, Modulus of Elasticity)	Positive	Allows reduction in resin, maintaining board properties
				Wet properties (Thickness swelling, Water absorption)	Positive	
				Formaldehyde content	Positive	
Waferboard	2	0.25–1.00%	10% paste	Mechanical properties (Internal Bond, Modulus of Rupture, Modulus of Elasticity)	Positive	Allows reduction in resin, maintaining board properties
				Wet properties (Thickness swelling, Water absorption)	Neutral	
				Formaldehyde content	Neutral	
Plywood	1	0.22%	2% suspension	Shear strength	Positive	
				Wood failure	Neutral	
	2	0.23–0.68%	10% paste	Shear strength	Neutral	Can substitute conventional thickeners, maintaining board properties
				Wood failure	Neutral	
